# Connections between Immune-Derived Mediators and Sensory Nerves for Itch Sensation

**DOI:** 10.3390/ijms222212365

**Published:** 2021-11-16

**Authors:** Sumika Toyama, Mitsutoshi Tominaga, Kenji Takamori

**Affiliations:** 1 Juntendo Itch Research Center (JIRC), Institute for Environmental and Gender-Specific Medicine, Juntendo University Graduate School of Medicine, 2-1-1 Tomioka, Chiba 279-0021, Japan; su-toyama@juntendo.ac.jp (S.T.); tominaga@juntendo.ac.jp (M.T.); 2 Anti-Aging Skin Research Laboratory, Juntendo University Graduate School of Medicine, 2-1-1 Tomioka, Chiba 279-0021, Japan; 3 Department of Dermatology, Juntendo University Urayasu Hospital, 2-1-1 Tomioka, Chiba 279-0021, Japan

**Keywords:** cytokines, immune cell, itch mediator and modulator, sensory neuron

## Abstract

Although histamine is a well-known itch mediator, histamine H_1_-receptor blockers often lack efficacy in chronic itch. Recent molecular and cellular based studies have shown that non-histaminergic mediators, such as proteases, neuropeptides and cytokines, along with their cognate receptors, are involved in evocation and modulation of itch sensation. Many of these molecules are produced and secreted by immune cells, which act on sensory nerve fibers distributed in the skin to cause itching and sensitization. This understanding of the connections between immune cell-derived mediators and sensory nerve fibers has led to the development of new treatments for itch. This review summarizes current knowledge of immune cell-derived itch mediators and neuronal response mechanisms, and discusses therapeutic agents that target these systems.

## 1. Introduction

Itch (or pruritus) is an unpleasant sensation inducing the desire to scratch [1], as well as being a major and distressing symptom of many skin and systemic diseases. Chronic itch represents a significant clinical problem resulting from renal [2], liver [3], and bowel diseases [4], as well as several serious skin diseases, such as atopic dermatitis (AD). Histamine is one of the best-evaluated itch mediators. If an itch is caused by histamine, antihistamines (histamine H_1_-receptor blockers) can be used to control it. However, recent studies have suggested that histamine-independent pathways are involved in chronic itch, making antihistamines ineffective in the treatment of these patients [5,6,7]. Thus, the mechanisms of itch development and enhancement other than through histamine remain to be determined. Analyses of the interactions between immune cells and sensory neurons have shown that cytokines produced by immune cells during inflammation enhance itch, and that they act directly on sensory nerve fibers to induce and/or sensitize itch sensation.

This review focuses on immune cell-derived itch mediators and describes the mechanisms by which they connect to sensory nerves to produce and enhance itch.

## 2. Subtype of Sensory Neurons

Generally, itch sensation is generated by the binding of itch-inducing substances (pruritogens) to their cognate receptors (pruriceptors) on peripheral sensory afferents, especially unmyelinated C-fibers [8]. Single-cell RNA-seq has classified the sensory neuron system into five neurofilament (NF)-containing clusters, two peptidergic (PEP) nociceptor clusters, a tyrosine hydroxylase (TH)-containing cluster and three non-peptidergic (NP) nociceptor clusters [9]. The NF clusters were shown to express neurofilament heavy chain (*Nefh*) and parvalbumin (*Pvalb*), molecules previously associated with myelinated dorsal root ganglion (DRG) neurons. The PEP clusters were found to express substance P (SP, also known as *Tac1*), TRKA (*Ntrk1*) and calcitonin gene-related peptide (CGRP, also known as *Calca*), molecules previously associated with peptidergic nociceptors. The TH cluster showed distinct expression of tyrosine hydroxylase (*Th*), which is also expressed in a distinct subclass of unmyelinated neurons. Finally, the NP clusters were found to express Mas-related G protein coupled receptor D (*Mrgprd*) and *P2rx3*, molecules previously associated with nonpeptidergic nociceptors. Notably, NP clusters express receptor genes for itch mediators.

**Figure 1:**
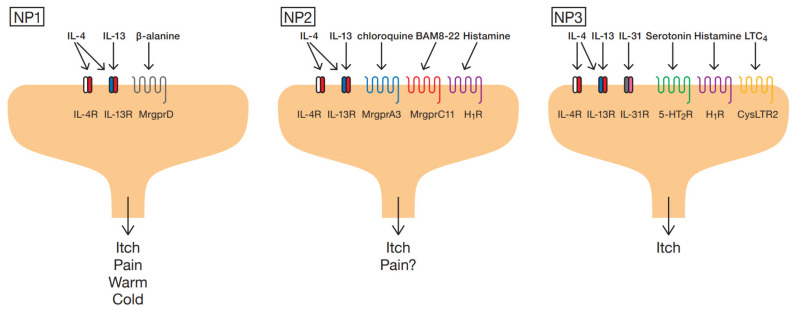
NP clusters of itch-related sensory nerves and itch-related receptors expressed on them. NP1 neurons are positive for IL-4Rα, IL-13 Rα and MrgprD (**left**). NP2 neurons are positive for IL-4Rα, IL-13 Rα, MrgprA3, MrgprC11 and H_1_R (**middle**). NP3 neurons are positive for IL-4Rα, IL-13 Rα, IL-31R, 5-HT2R, H_1_R and CysLTR2 (**right**).

NP1 expresses the β-alanine receptor *Mrgprd* [10] and the lysophosphatidic acid receptors *Lpar3* and *Lpar5*. Chloroquine (CQ) receptor (*Mrgpra3*) and bovine adrenal medulla (BAM) 8–22 receptor (*Mrgprx1*:human, *Mrgprc11*:mice) [11] are expressed on NP2; whereas the interleukin (IL)-31 receptor *Il31ra*, the oncostatin M receptor *(OSM)*, the leukotriene (LT) C_4_ receptor *Cysltr2* [12] and the serotonin receptors *Htr1f* and *Htr2a* are expressed on NP3. Histamine receptor (*H1R*) was detected on NP2 and NP3 [9] (Figure 1). In addition, NP1, NP2, and NP3 were found to be more enriched in neurons that express *Il4ra* and *Il13ra1* than in other types of neurons such as NF and PEP [13].

## 3. Itch Mediators and Modulators from Immune Cells

**Figure 2:**
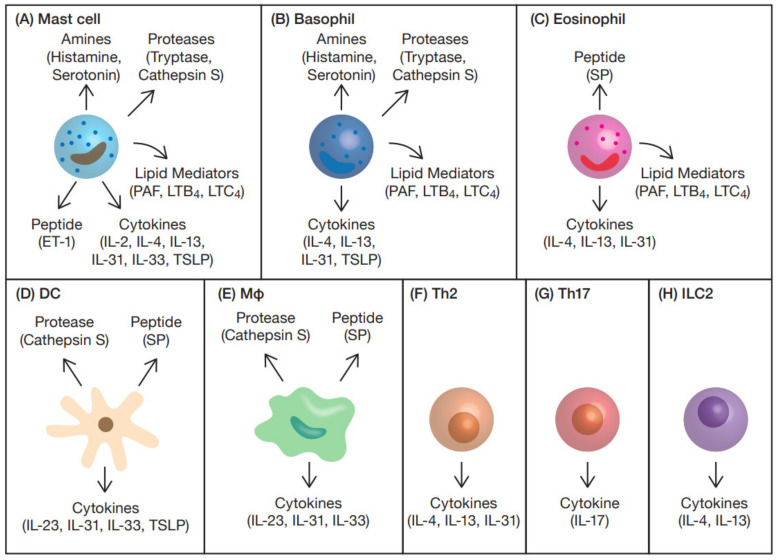
Immune cells and itch mediators and modulators. (**A**) Mast cells produce amines (histamine and serotonin), proteases (tryptase and cathepsin S), peptide (ET-1), cytokines (IL-2, IL-4, IL-13, IL-31, IL-33 and TSLP) and lipid mediators (PAF, LTB_4_, and LTC_4_). (**B**) Basophils produce amines (histamine and serotonin), proteases (tryptase and cathepsin S), cytokines (IL-4, IL-13, IL-31 and TSLP) and lipid mediators (PAF, LTB_4_ and LTC_4_). (**C**) Eosinophils produce peptide (SP), cytokines (IL-4, IL-13 and IL-31) and lipid mediators (PAF, LTB_4_, LTC_4_). (**D**) DCs produce protease (cathepsin S), peptide (SP) and cytokines (IL-23, IL-31, IL-33 and TSLP). (**E**) Macrophages produce protease (cathepsin S), peptide (SP) and cytokines (IL-23, IL-31 and IL-33). (**F**) Th2 cells produce cytokines (IL-4, IL-13 and IL-31). (**G**) Th17 cells produce the cytokine IL-17. (**H**) ILC2 cells produce cytokines (IL-4 and IL-13).

Table 1 and Table 2 summarize the immune cell-derived itch mediators and modulators, and the therapeutic agents that target them. This section describes the itch mediators and modulators produced by immune cells. As detailed above, the primary sensory nerves associated with itch have been classified into at least three subtypes, each of which has its own response profile. Based on the subtypes of nerve cells, the itch mediators and modulators derived from immune cells are also summarized (Figure 2).

### 3.1. Amines

#### 3.1.1. Histamine

Histamine, the most well-known pruritogen, is produced by mast cells, basophils and keratinocytes [14,15,16,17,18,19,20,21]. Histamine evokes itch via histamine H_1_ and H_4_ receptors [19,22]. Histamine H_1_ receptor (H_1_R) is a G protein-coupled receptor (GPCR) [20,23,24,25], a class of receptors globally expressed in various tissues, including sensory nerves [17,21]. Histamine H_4_ receptor (H_4_R) is also a GPCR [20,24,25] and is mainly expressed in immunocompetent cells, including mast cells, eosinophils, neutrophils, monocytes, dendritic cells (DCs) and T cells; as well as in intestinal epithelia, spleen, lung, synovial tissue, the central nervous system (CNS), sensory neurons, and cancer cells [21,24,26]. H_1_R and H_4_R on histaminergic nerves bind histamine and then activate transient receptor potential vanilloid (TRPV) 1 [17,27]. The H_4_R antagonist, ZPL-3893787, improved AD symptoms including itch [28].

A H_3_R inverse agonist was found to induce strong itch in mice. This H_3_R inverse agonist induced pruritus but could be completely blocked by combined treatment with an H_1_R and an H_4_R antagonist, whereas the H_2_R antagonist failed to inhibit the scratch response. The decreased threshold in response to H_3_R antagonism is thought to activate H_1_R and H_4_R on sensory neurons, leading to the excitation of histamine-sensitive afferents and eliciting a sensation of itch [29].

#### 3.1.2. Serotonin

Serotonin (5-hydroxytryptamine; 5-HT), which is produced by mast cells, basophils and platelets [15,30,31,32,33], evokes scratching in rodents via the 5-HT_2_ receptor, which is TRPV4-dependent [34,35,36,37]. The 5-HT_2_ receptor is expressed in immunocompetent cells, including macrophages, DCs, Langerhans cells, CD3^+^ T cells, melanocytes, vascular smooth muscle cells, endothelial cells, central and peripheral neurons including primary sensory neurons (DRG/trigeminal ganglion cells) [31,38,39,40]. Activation of the 5-HT_2_ receptor, which belongs to the GPCR super-family and is coupled to the Gq/11 protein, leads to increases in inositol trisphosphate (IP3) and diacylglycerol (DG) levels, generating an antinociceptive effect [38,40]. Sertraline, a selective serotonin reuptake inhibitor, has been found to be effective in treating serotonin-targeted itch [41]. In addition, existing drugs, such as the selective 5-HT_2_ receptor antagonist sarpogrelate, may have expanded future clinical application in the treatment of itch.

### 3.2. Proteases

#### 3.2.1. Tryptase

Tryptase, a serine protease with trypsin-like specificity, consists of seven distinct isoforms, α, βI, βII, βIII, δ, ε and γ, encoded by a set of protease genes clustered together on chromosome 16p13.3 [42,43,44,45]. The tryptase best characterized to date is β-tryptase, and the term “tryptase” is often used as a synonym for this molecule [45]. Tryptase is expressed in mast cells and basophils [45,46,47,48,49]. Intradermal injection of tryptase elicits scratching in mice [50]. Proteases, including tryptase, activate protease-activated receptors (PARs) by cleaving a part of their extracellular domain. PARs are GRCRs, characterized by a unique mechanism of self-activation following specific proteolytic cleavage of their extracellular domains. To date, four PARs have been identified, PAR-1, PAR-2, PAR-3, and PAR-4, which are encoded by the *F2R*, *F2RL1*, *F2RL2*, and *F2RL3* genes, respectively [51,52]. PAR-2 is activated by trypsin-like serine proteases and is widely distributed throughout the mammalian body. In the skin, PAR-2 is expressed by almost all cell types, especially by keratinocytes. In addition, endothelial cells, fibroblasts, sensory neurons, and inflammatory cells such as mast cells, T lymphocytes, eosinophils, neutrophils, monocytes, macrophages, and DCs have been reported to express functional PAR-2 [52]. Tethered ligands, such as the PAR-2 agonist SLIGRL-NH_2_, have been shown to elicit scratching in mice, but not rats [53]. Activated PAR-2 coactivates TRPV1 channels stimulating the release of the neuropeptides SP and CGRP from nerve terminals [54,55]. In addition, SLIGRL-NH_2_ enhances CQ- and BAM8-22-induced itch and acts as a modulator [56].

#### 3.2.2. Chymase

Chymase is a chymotrypsin-like serine endopeptidase stored in mast cell secretory granules [18]. Human chymase, encoded by the *CMA1* gene located on chromosome 14q11.2, co-localizes with clusters formed by cathepsin G, granzyme B and granzyme C/H [46,57,58]. In rats, the chymase-encoding gene is located on chromosome 15p12/13, and in mice on chromosome 14C1/2 [58,59,60,61,62]. Chymase also activates PAR-2 [63,64]. The chymase specific inhibitor Y-40613 was found to suppress scratching behavior in a mouse model of pruritus [65]. In the eyes, chymase also induced scratching behavior, which was suppressed by the selective chymase inhibitor ONO-WH-236 [64].

#### 3.2.3. Cathepsin S

Cathepsin S is a cysteine protease produced by DCs, macrophages, basophils and keratinocytes [19,66,67]. Cathepsin S activates PAR-2, PAR-4 and MrgprC11 to produce itch [68,69,70]. Intradermal injection of the selective PAR-4 agonist AYPGKF-NH_2_ (AYP) elicited scratching behavior in mice [56,71], which was prevented by the selective PAR-4 antagonist (pepducin P4pal-10) [71]. AYP-induced itch was reduced by gastrin-releasing peptide (GRP), NK-1, TRPV1 and a TRPA1 antagonist. These results indicated that PAR-4-activated itch is induced via TRPV1/TRPA1 in mice [71]. Moreover, touch-evoked scratching (alloknesis) was observed following intradermal injection of AYP, but not PAR-2 [56]. Cathepsin S also evoked a calcium response in mouse DRG neurons, which is reduced by PAR-2 antagonists and in TRPV1-/-or TRPA1-/-mouse-derived DRGs. In addition, intradermal injection of cathepsin S induced scratching behavior, which was inhibited by the cathepsin S inhibitor LHVS [70].

### 3.3. Peptides

#### 3.3.1. Substance P

Substance P (SP) is a short neuropeptide of the tachykinin family, consisting of 11 amino acids (Arg-Pro-Lys-Gln-Gln-Phe-Phe-Gly-Leu-Met-NH_2_), and is one of most potent pruritogens identified to date [72,73]. SP is expressed by many cell types, including sensory neurons, astrocytes, microglia, epithelial cells, endothelial cells and immune cells, including T cells, macrophages, DCs and eosinophils [11,20,74]. SP binds to neurokinin 1 receptor (NK-1R) and another class of receptors involved in itch signaling, consisting of mouse MrgprA1, mouse MrgprB2 and human MrgprX2. NK-1R is a tachykinin receptor belonging to the GPCR family and expressed in the CNS, keratinocytes, fibroblasts and mast cells [72,73]. In humans, SP promotes degranulation by binding to mast cell NK-1R, releasing histamine and LTB_4_ and causing itch [22,73]. In mice, SP induces itch through direct action on primary sensory neurons, as well as by release of nitric oxide (NO) and LTB_4_ from keratinocytes, rather than by mast cell degranulation [22,75].

#### 3.3.2. Endothelin-1

Endothelin (ET)-1 is a 21 amino-acid peptide member of the endothelin family and a potent pruritogen that can elicit scratching at low concentration (10–400 pmol/site) [76,77,78]. ET-1 is produced by mast cells, endothelial cells and keratinocytes in the skin [54,76,77,78]. ETs have two active receptors, ET_A_ and ET_B_, which belong to the GPCR superfamily [78,79,80]. Endothelin receptors are widely expressed in all tissues [81], and ET-1-evoked scratching is mediated by ET_A_ [76]. In addition, the endothelin receptor antagonist bosentan inhibited symptoms including itch in AD model mice [82].

### 3.4. Cytokines

#### 3.4.1. IL-2

Interleukin (IL)-2 is a 15.5 kDa cytokine secreted by antigen-activated CD4^+^ T cells and mast cells [83,84,85,86]. It was first described as a T cell growth factor and later also found to have the ability to act on natural killer (NK) cells and NKT cells, to activate B cells, and to induce the proliferation of regulatory T cells (Tregs), innate lymphoid cells (ILCs) and effector T cells. IL-2 has three receptors, each of which is composed of three subunits: IL-2 receptor α (IL-2Rα, CD25), IL-2Rβ (CD122), and IL-2Rγ (CD132). IL-2Rα is expressed by several types of immune cells, including Tregs, ILC2, activated CD4^+^ and CD8^+^ T cells, B cells, CD56^hi^ NK cells, mature DCs, and endothelial cells. IL-2Rβ is mainly expressed by multiple lymphoid populations, such as Tregs, memory CD8^+^ T cells, NK cells, and NKT cells, and to some extent, by monocytes and neutrophils. IL-2Rγ is expressed mostly by hematopoietic cells [83,86,87]. The binding of IL-2 to its receptors induces trans-phosphorylation of Janus kinase (JAK) 1 and JAK3. This, in turn, activates the JAK/signal transducer and activator of transcription (STAT), phosphoinositide (PI) 3-kinase and MAPK signaling pathways [86,87]. Intravenous IL-2 treatment has been approved for the treatment of patients with metastatic melanoma and renal cell carcinoma, with beneficial results in a subset of patients, although severe pruritus is a known side effect [83,86,87,88,89]. Moreover, intradermal injection of IL-2 in either healthy subjects or patients with AD induced pruritus and erythema [89,90]. The calcineurin inhibitor cyclosporine A has been shown to downregulate IL-2 synthesis, reducing pruritus in patients with treatment resistant Sezary syndrome, as well as in patients with AD [89].

#### 3.4.2. IL-4

IL-4 is a type 2 cytokine produced by T helper (Th) 2 cells, lymph node T follicular helper (Tfh) cells, mast cells, basophils, eosinophils and ILC2 [91,92,93,94]. IL-4 has two receptors, IL-4Rα (CD124) and the more common IL-4Rγ, with IL-4 having higher affinity to IL-4Rα [95]. IL-4 signals through the IL-4Rα/γC complex in hematopoietic cells, such as lymphocytes and DCs. IL-4 binds IL-4Rα/γC and activates the downstream signaling molecules JAK1/JAK3 and then STAT6. Non-hematopoietic cells including keratinocytes also express IL-4Rα/IL-13Rα1 complex, which binds both IL-4 and IL-13, resulting in the downstream activation of JAK1/TYK2/JAK2 and then STAT6/STAT3 [93]. IL-4-evoked mouse DRG neurons respond to calcium, and deletion of IL-4Ra on sensory neurons was found to disrupt scratching behavior in a mouse model of AD. Moreover, IL-4 has been suggested as a modulator of itch because it enhances itch by increasing the neural responses induced by histamine, chloroquine, TSLP, and IL-31 [13,91]. Intradermal administration of IL-4 has also been reported to induce itching and alloknesis [96,97]. Dupilumab, a monoclonal antibody that binds specifically to the shared alpha chain subunit of the IL-4 and IL-13 receptors, was associated with improvements in clinical end points, including reduced pruritus in AD [98].

#### 3.4.3. IL-13

IL-13 is another type 2 cytokine produced by Th2, ILC2, mast cells, basophils, and eosinophils [91,92,93,94]. It has two receptors, IL-13Rα1 (CD213α1) and IL-13Rα2 (CD213α2). IL-13Rα1 alone binds IL-13 with low affinity, but when paired with IL-4Rα it binds IL-13 with high affinity and forms a functional IL-13 receptor that signals and results in activation of STAT3/6 [93,99]. Similar to IL-4, intradermal administration of IL-13 has been reported to induce itching and alloknesis [96,97].

To date, the role of IL-13Rα2 in itch has been unclear. However, a recent study showed that the expression of IL-13Rα2 is upregulated in the skin of patients with AD, but not in the skin of patients with psoriasis, in a disease activity-dependent manner. In keratinocytes, IL-13 regulated IL-13Rα2 expression level and promoted IL-13Rα2 signaling. In addition, TLR2 activation was found to increase IL-13 mediated itch by potentiating IL-13Rα2, suggesting that IL-13Rα2 signaling promotes AD symptoms including itch [100]. Monoclonal antibodies that target and neutralize IL-13, Tralokinumab and Lebrikizumab, both improved AD symptoms including itch [28].

#### 3.4.4. IL-17

IL-17A, also called IL-17, is produced by various cell types of T cells, including the Th17 subset of CD4^+^ T cells, CD8^+^ T cells, γδ T cells, and NKT cells, as well as by immune cells such as lymphoid tissue inducer (LTi)-like cells and neutrophils, and nonimmune cells such as Paneth cells. IL-17 has two receptors, IL-17RA and IL-17RC, which form a heterodimer. Binding of IL-17A or an IL-17F heterodimer to IL-17R induces Act1 activation, which, in turn, activates multiple signaling cascades that operate through different TNF receptor-associated factor (TRAF) proteins. Subsequently, the complex associates with TRAF6, leading to the activation of NF-kB, MAPK-AP-1, and C/EBP. ERK1/2 mediates the phosphorylation of C/EBPβ at Thr188, with the CBAD of IL-17R also required for IL-17-mediated inducible phosphorylation of C/EBPβ at Thr179 through GSK3β. IL-17 can also induce different feedback regulatory responses by inducing and/or recruiting deubiquitinase enzymes (A20 and USP25) or kinases (TBK1) [101,102]. Three randomized, controlled, phase 3 trials reported that brodalumab, an IL-17 receptor A antagonist, is safe and effective in treating moderate-to-severe psoriasis. In addition, brodalumab demonstrated improved itch responses in psoriasis [103]. These results suggest that IL-17 may act as an itch mediator and/or modulator. Other studies, however, have reported that IL-17 is neither a mediator nor a modulator of itching [104], leading to the need for additional research.

#### 3.4.5. IL-23

IL-23 belongs to the IL-12 family of proinflammatory cytokines. IL-23 is heterodimeric, being composed of IL-12p40 and p19 molecules. It is produced by activated DCs and macrophages in response to microbial pathogens, with its production enhanced by interactions between the costimulatory molecule CD40 and its ligand. IL-23 signals via IL-12Rβ1 and IL-23R and mediates the phosphorylation of STAT3 and STAT4 by JAK2 and Tyk2 [105,106]. Intradermal injection of IL-23 did not induce scratching behavior, but calcium imaging showed that about 5% of DRG neurons in mice responded to IL-23. IL-23 was also found to attenuate histamine-induced itch, suggesting that this cytokine may function as a desensitizer [104]. In addition, IL-23 might play a role in regulating histaminergic itch by modulating TRPV1 activity [104].

#### 3.4.6. IL-31

IL-31, which belongs to the IL-6 family of cytokines, is produced by Th2 cells, mast cells, eosinophils, basophils, macrophages and DCs [19,89,107,108,109,110,111]. IL-31 binds to its receptor, a complex composed of IL-31 receptor A (IL-31RA) and oncostatin M (OSM) receptor, which is expressed on keratinocytes, epithelial cells, mast cells, basophils, eosinophils, macrophages, sensory neurons, DRG and the dorsal horn of the spinal cord [13,89,91,111]. IL31RA/OSMR is activated with similar affinities by OSM and IL-31. Binding of IL-31 leads to activation of diverse kinase pathways, including the JAK1/2/STAT3, ERK1/2, PI3K/Akt, p38 MAPK and JNK cascades [111,112,113,114]. *IL-31*Tg mice showed a marked and significant increase in cutaneous nerve fiber density in lesional skin compared with uninvolved or healthy skin [115]. In addition, cutaneous and intrathecal injections of IL-31 evoked intense itch, which was TRPV1 and TRPA1-dependent [113]. Moreover, a more recent study showed that transmembrane protein 184B (TMEM184B) is necessary for IL-31-induced itch [116]. Thus, the details of the mechanism of IL-31-induced itch are becoming clearer, and target molecules that can lead to treatment are being identified one after another.

### 3.5. Lipid Mediators

#### 3.5.1. PAF

Platelet-activating factor (PAF) is produced and released by mast cells, basophils, neutrophils, eosinophils, monocytes, macrophages, fibroblasts, platelets, endothelial cells, and cardiac muscle cells, all of which play important roles in inflammatory and thrombotic diseases. PAF is an inflammatory factor and has important functions in acute and chronic inflammation [117,118]. PAF receptor (PAFR) has been found in a host of cell membranes, including those of platelets, neutrophils, macrophages, mononuclear leukocytes, and eosinophils, as well as on hippocampal nerves, microglia, astrocytes, and oligodendrocyte progenitor cells [118]. Intradermal PAF injection evoked scratching behavior [35,119] and induced histamine release through degranulation of mast cells, contributing to itch accompanied by flare and wheal reactions [120].

#### 3.5.2. LTB_4_

Leukotrienes (LTs) are eicosanoid lipid mediators generated upon activation of both immune and structural cells such as epithelial cells and endothelial cells. LTB_4_, a 5-lipoxygenase metabolite, is increased in the skin of AD model mice [121]. This molecule is produced and released by various types of immune cells, including mast cells, basophils, eosinophils, and macrophages [122,123,124,125]. LTB_4_ has two receptors, BLT1 and BLT2, which are GPCR and present on cell surfaces, with BLT1 having higher affinity and activity than BLT2. BLT1 is mainly expressed by leukocytes and DRG neurons, whereas BLT2 is expressed on many tissues [126,127,128]. LTB_4_-induced DRG neurons respond to calcium, an effect inhibited by the LTB_4_ antagonist ONO-4057 [128]. Intradermal LTB_4_ injection induces scratching via TRPA1 and TRPV1 [129]. Moreover, the LTB_4_ receptor antagonist CMHVA attenuated IL-31-induced scratching [130].

#### 3.5.3. LTC_4_

LTC_4_ is a cysteinyl LT produced and released by mast cells, basophils, and eosinophils [131,132,133]. Its receptors, CysLTR1 and CysLTR2, are widely expressed by hematopoietic and structural cells [12]. Basophils have been shown to release LTC_4_ upon stimulation with antigen-specific IgE, which binds to CysLTR2 expressed on sensory nerve fibers (mainly NP3 nerves), evoking acute severe itch (itch flares) of AD [132]. Moreover, the LTC4/CysLTR2 pathway was shown to contribute not only to acute but also to chronic itch [12].

### 3.6. Others

#### 3.6.1. IL-33

IL-33, a member of the IL-1 cytokine family, is considered important for host defenses and allergy by inducing Th2 cytokine production via the IL-33 receptor. This receptor is a heterodimer composed of IL-1 receptor-like 1 (IL-1RL1; also called ST2) and IL-1 receptor accessory protein (IL-1RAcP) molecules. IL-33 was first identified as a nuclear protein expressed in endothelial cell nuclei and was shown to be constitutively expressed in the nuclei of various cell types, such as endothelial and epithelial cells [134,135]. IL-33 was also recently shown to be constitutively expressed in other cells, including DCs, macrophages, mast cells, fibroblasts, smooth muscle cells, platelets and megakaryocytes [135,136]. ST2 expressing cells include basophils, mast cells, eosinophils, macrophages, DCs, NK cells, NKT cells, Th2 cells, cytotoxic T cells, Tregs, B cells, ILCs, microglia, astrocytes, neurons, epithelial cells, endothelial cells, and fibroblasts [135,137,138]. Treatment of AD model mice with anti-IL-33 antibody improved AD-like symptoms, including scratching behavior [139]. Moreover, IL-33/ST2 signaling was found to mediate chronic itch in a mouse model of contact hypersensitivity through the astrocytic JAK2/STAT3 cascade [140]. IL-33 was also shown to evoke calcium responses in neurons, with enhanced CQ evoking calcium responses [138]. Taken together, these findings suggested that IL-33 also functions as a modulator to enhance itch.

#### 3.6.2. TSLP

Thymic stromal lymphopoietin (TSLP) is a IL-7 like cytokine belonging to the IL-2 cytokine family [110,141]. It is primarily produced by epithelial cells, including keratinocytes, fibroblasts and stromal cells, as well as by DCs, mast cells, and basophils [110,142]. Its receptor, TSLPR, is expressed on monocytes/macrophages, T cells, B cells, mast cells, eosinophils, NK cells, DCs, keratinocytes and sensory neuronal endings [143,144,145,146,147,148]. TSLPR is activated upon binding of TSLP, which activates JAK1/2 and STAT1/3/4/5/6 [149,150]. Intradermal injection of TSLP evoked scratching behavior. This is initiated by the binding of TSLP to TSLPR expressed on sensory nerve fibers. The TSLP-induced itch also required TRPA1, with the expression and release of keratinocyte-derived TSLP depending on the ORAI1/NFAT calcium signaling pathway [148]. Epithelial cell-derived cytokines, including TSLP and IL-33, strongly activate ILC2 and recruit Th2 cells into the skin. ILC2 and Th2 cells are rich sources of type 2 cytokines, which can initiate and perpetuate allergic skin inflammation, including itch, by recruiting basophils and eosinophils [91].

## 4. Immune System-Targeted Antipruritic Drugs

### 4.1. Therapeutic Drugs for Amines

As described above, conventional treatments such as anti-histamines are often ineffective in patients with chronic pruritus. Therapeutic drugs other than antihistamines that target histamine consist of topical or systemic anti-inflammatory and immunomodulatory agents (e.g., cyclosporine A, pimecrolimus, tacrolimus and corticosteroids) [6]. Serotonin-targeted itch treatments include sertraline [41], but the clinical application of existing drugs such as sarpogrelate may also expand in the future.

### 4.2. Therapeutic Drugs for Proteases

Protease-targeted therapies for itch are thought to be similar to histamine [6]. Furthermore, the selective chymase inhibitor ONO-WH-236 and the cathepsin S inhibitor LHVS were found to suppress scratching behavior [64,70]. In the future, protease inhibitors may become a more established method of treating itch.

### 4.3. Therapeutic Drugs for Peptides

Gabapentin, pregabalin and capsaicin are effective for the treatment of neuropathic itch [6]. A phase II randomized clinical trial showed that a NK-1R (a receptor for SP) inhibitor was effective for treating itch in patients with psoriasis [151].

### 4.4. Therapeutic Drugs for Cytokines

More recently, a variety of monoclonal antibodies have been shown to be effective in the treatment of itch. For example, dupilumab was found to improve AD symptoms and itch [152]. Most cytokines are activated via JAK/STAT signaling. Recently, a JAK inhibitor, delgocitinib, was reported to improve symptoms and itching of AD and was approved in Japan [153]. Moreover, Baricitinib, which inhibits JAK1 and JAK2, and Abrocitinib, which inhibits JAK1, improved AD symptoms including itch [28]. JAK inhibitors will be used for the treatment of itch in AD in the future.

### 4.5. Therapeutic Drugs for Lipid Mediators

CMHVA, a LTB_4_ receptor antagonist, was found to improve itch [130], suggesting it may be targeted as a lipid mediator to treat itch in the future.

**Table 1:** Immune cell-derived itch mediators and therapeutic methods.

Category	Pruritogens	Receptors	Therapeutic Methods	Reference
Amines	Histamine	H_1_R/H_4_R	Anti-histamine/Anti-inflammatory, immuno-modulatory topical and systemic therapy (Cyclosporine A, Pimecrolimus, Tacrolimus and Corticosteroids)	[6,28]
	Serotonin	5-HT_2_ receptor	Sertraline	[41]
Proteases	Tryptase	PAR-2	Anti-histamine/Cyclosporine A/Pimecrolimus/Tacrolimus/Corticosteroids	[6]
	Chymase	PAR-2	ONO-WH-236/Anti-histamine/Cyclosporine A/Pimecrolimus/Tacrolimus/Corticosteroids	[6,63]
	Cathepsin S	PAR-2/PAR-4	LHVS/Anti-histamine/Cyclosporine A/Pimecrolimus/Tacrolimus/Corticosteroids	[6,70]
Peptides	Substance P	NK-1R	Serlopitant/Gabapentin/Pregabalin/Capsaicin	[6,151]
	Endothelin-1	ET_A_	Bosentan	[82]
cytokines	IL-2	IL-2R	Cyclosporine A/Delgocitinib/Baricitinib/Abrocitinib	[28,86,87,89,153]
	IL-4	IL-4Rα/γC	Dupilumab/Delgocitinib/Baricitinib/Abrocitinib	[28,93,99,152,153]
		IL-4Rα/IL-13Rα1		
	IL-13	IL-4Rα/IL-13Rα1	Dupilumab/Tralokinumab/Lebrikizumab	[28,93,99]
	IL-17	IL-17RA/IL-17RC	Brodalumab	[103]
	IL-23	IL-12Rβ1/IL-23R	Delgocitinib/Baricitinib	[28,105,106,153]
	IL-31	IL-31RA/OSMR	Nemolizumab/Delgocitinib/Baricitinib/Abrocitinib	[28,111,112,113,114,153,154]
	IL-33	ST2/IL-1RAcP	Etokimab/Delgocitinib/Baricitinib	[28,140,153]
	TSLP	TSLPR	Tezepelumab/Delgocitinib/Baricitinib/Abrocitinib	[28,149,150,153,155]
Lipid mediators	PAF	PAFR	PAF antagonist	[118,156]
	LTB_4_	BLT1/BLT2	CMHVA	[128,130]
	LTC_4_	CysLTR1/CysLTR2	CysLTR2 antagonist	[157]

**Table 2:** Itch modulators from immune cells.

Ligands	Receptors	Source	Modulation
SLIGRL-NH_2_	PAR-2	mast cells, basophils	Enhances CQ and BAM8-22 induced itch
IL-4	IL-4Rα/γC IL-4Rα/IL-13Rα1	Th2, Tfh, ILC2, mast cells, basophils, eosinophils	Enhanced neuronal responsiveness to histamine, CQ, TSLP and IL-31
IL-13	IL-13Rα1/IL-13Rα2	Th2, ILC2, mast cells, basophils, eosinophils	May enhance neuronal responsiveness to histamine, CQ, TSLP and IL-31, as well as IL-4
IL-23	IL-12Rβ1/IL-23R	DCs, macrophages	Reduced histamine-induced itch
IL-33	ST2/IL-1RAcP	DCs, macrophages, mast cells	Enhanced CQ evoked calcium responses

## 5. Conclusions

This review presents recent knowledge regarding immune cell-derived mediators and modulators of itch. Many of these mediators cause nerve firing via their respective receptors expressed on sensory nerves, affecting the induction and modulation of itch. The variety of immune-derived itch mediators alone suggests that the mechanisms of itch are diverse. Although it is practical to focus on a common molecule such as JAK as a therapeutic target for itch, in fact, the development of therapeutic agents that target individual itch mediators and their receptors is ongoing. Thus, in clinical practice, however, in the future, due to the diverse molecules involved, a combination of therapies may be required to treat itch. It would be ideal to develop a system to test for itch mediators in each individual patient to determine the best treatment or appropriate combination therapy for each individual patient.
